# Down-Regulation of EBV-LMP1 Radio-Sensitizes Nasal Pharyngeal Carcinoma Cells via NF-κB Regulated ATM Expression

**DOI:** 10.1371/journal.pone.0024647

**Published:** 2011-11-09

**Authors:** Xiaoqian Ma, Lifang Yang, Lanbo Xiao, Min Tang, Liyu Liu, Zijian Li, Mengyao Deng, Lunquan Sun, Ya Cao

**Affiliations:** 1 Cancer Research Institute, Xiangya School of Medicine, Central South University, Changsha, China; 2 Center for Molecular Medicine, Central South University, Xiangya Hospital, Changsha, China; 3 Key Laboratory of Carcinogenesis and Invasion, Ministry of Education, Key Laboratory of Carcinogenesis, Ministry of Health, Changsha, China; 4 Center for Molecular Imaging, Central South University, Changsha, China; University of Windsor, Canada

## Abstract

**Background:**

The latent membrane protein 1 (LMP1) encoded by EBV is expressed in the majority of EBV-associated human malignancies and has been suggested to be one of the major oncogenic factors in EBV-mediated carcinogenesis. In previous studies we experimentally demonstrated that down-regulation of LMP1 expression by DNAzymes could increase radiosensitivity both in cells and in a xenograft NPC model in mice.

**Results:**

In this study we explored the molecular mechanisms underlying the radiosensitization caused by the down-regulation of LMP1 in nasopharyngeal carcinoma. It was confirmed that LMP1 could up-regulate ATM expression in NPCs. Bioinformatic analysis of the ATM ptomoter region revealed three tentative binding sites for NF-κB. By using a specific inhibitor of NF-κB signaling and the dominant negative mutant of IkappaB, it was shown that the ATM expression in CNE1-LMP1 cells could be efficiently suppressed. Inhibition of LMP1 expression by the DNAzyme led to attenuation of the NF-κB DNA binding activity. We further showed that the silence of ATM expression by ATM-targeted siRNA could enhance the radiosensitivity in LMP1 positive NPC cells.

**Conclusions:**

Together, our results indicate that ATM expression can be regulated by LMP1 via the NF-κB pathways through direct promoter binding, which resulted in the change of radiosensitivity in NPCs.

## Introduction

Radio-resistance has been one of the impediments in clinical settings for effective cancer therapy, which is thought to be associated with multiple signaling pathways in different cancer types. ATM (ataxia telangiectasia mutated) is a nuclear 350-kDa protein kinase with a carboxylterminal phosphatidylinositol 3-kinase-like kinase domain [1]. It functions as a member of a coordinated system that detects DNA breaks; arrests the cells temporarily at G1, S, or G2 checkpoints; and activates DNA repair [2]. Cells lacking functional ATM protein show increased sensitivity to ionizing radiation (IR) and other genotoxic events [3], [4], [5]. NF-κB (nuclear factor kappa B) can activate a great number of genes involved in stress responses, inflammation, and programmed cell death (apoptosis) [6]. P50 homodimers or p50/p65 or p50/c-Rel heterodimers bind to the NF-κB DNA binding sites in the promoter regions of many stress-responsive genes, suggesting a complex gene and physiological regulation network controlled by NF-κB in stress response [7]. The elevated basal NF-κB activity in certain cancers has been linked to tumor resistance to chemotherapy and radiation [8]. Inhibition of NF-κB blocked the adaptive radioresistance [9]. Human breast cancer cells treated with fractional γ-irradiation displayed an enhanced clonogenic survival and NF-κB activation [10], [11]. Thus, it is logical to speculate that there could be a link between the ATM expression and NF-κB signaling, yet to be experimentally proven.

LMP1 (Latent Membrane Protein 1) is an Epstein–Barr virus encoded oncogenic protein composed of a short intracellular N terminus, six hydrophobic transmembrane domains, and an intracellular C terminus including three functional domains, CTAR1, CTAR2, and CTAR3. LMP1 activates its target genes via different signaling pathways that include NF-κB, JNK/c-Jun/AP-1, p38-MAPK/ATF, and JAK/STAT [12], [13], [14], [15], [16], [17]. Activation of NF-κB by LMP1 has been linked to the upregulation of some cellular proteins. Previously, we demonstrated that the phosphorothioate-modified “10–23” DNAzymes specifically targeted at the LMP1 mRNA could significantly down-regulate the expression of LMP1 in a nasopharyngeal carcinoma cell (NPC) and affected the down-stream pathways activated by LMP1, including the NF-κB pathway [18], [19]. It was also demonstrated that suppression of the LMP1 expression by the LMP1-targeted DNAzyme DZ1 could enhance radiosensitivity both in *vivo* and *vitro* [19]. To explore the molecular mechanism of the LMP1-DNAzyme mediated radiosensitization, bioinformatic analysis revealed there were three putative NF-κB binding sites in the ATM promoter region. Thus, we hypothesize that ATM expression can be regulated by LMP1 through the NF-κB pathways, which resulted in the change of radiosensitivity in NPCs. In the present study, we showed that LMP1 indeed activated ATM expression through the NF-κB pathway and inhibition of LMP1 expression by the DNAzyme attenuated the binding of the NF-κB transcription factor to the ATM promoter. Further evidence showed that the radiosensitivity was recovered when ATM expression was knocked down by siRNA in NPCs. Thus, the present studies support our hypothesis and provide further evidence for the use of LMP1-targeted DNAzymes as potential radiosensitizers for treatment of the EBV-associated carcinomas.

## Methods and Materials

### Cell lines and cell culture

CNE1 is a LMP1 negative and low differentiated nasopharyngeal squamous carcinoma cell line. The CNE1-LMP1 cell line constitutively expresses Epstein-Barr virus latent member protein-1(LMP1) and exhibits accelerated cell proliferation [20]. HNE2 is a EBV-LMP1-negative human nasopharyngeal carcinoma (NPC) cell line, HNE2-LMP1 is a cell line constantly expressing LMP1 after the introduction of full-length LMP1 cDNA into HNE2 cells [21]. HNE2-LMP1-DNMIκBα is a cell line constantly expressing dominant-negative mutant of IκBα (DNMIκBα) that had a deletion of 71 amino acids at the N terminus, which competitively inhibited the activation of NF-κB [22]. All the cell lines were grown in RPMI 1640 (GIBCO) supplemented with 10% fetal bovine serum, 1% glutamine, and 1% antibiotics and cultured at 37°C in a humidified incubator containing 5% CO_2_. The cells in logarithmic growth phase were used in all experiments.

### Plasmids

pSG5-B-346-LMP1 expresses the full-length LMP1 mRNA (the gift from Dr. Kenneth M, Lzumi. Brigham And Women's Hospital). To construct the ATM-promoter assay plasmid, the sequence of the full ATM promoter (Genbank accession# GXP_480587) was amplified from the genomic DNA of CNE1 cells with the forward primer of 5′-GGTACCTGCGTGGAGGATGGAGAAG-3′ and reverse primer of 5′- AGATCTAGAAGCCGCTGCGTTGCCT-3′. The 1233-bp product was cloned into KpnI and BglII sites of the pGL3 basic luciferase reporter vector (Promega).The resultant plasmid named pLuc-ATM. The corresponding mutants were derived from pLuc-ATM with substituting the sequence +105 CCGGGGAACTCCCTA +120 to +105 CCGAGAAACGCGCTA +120 (pLuc-ATMmNF1) and substituting the sequence +225CGGGCTTCCCCT+241 to +225CGAACTGTCTCT +241 (pLuc-ATMmNF2). Mutations at both sites were made to generate pLuc-ATMmNF1+2.

### DNAzyme and siRNA transfection

Prior to transfection, cells were seeded in 6-well plates overnight. The DNAzyme/TMP (tetra (4-methylpyridyl) porphyrine) mixtures were made at a charge ratio of 1 with 2 µM DNAzyme oligonucleotides as described [18], [23]. The mixtures were incubated for 15 min at room temperature to form the transfection complex. Cells were rinsed twice with PBS. The transfection mixtures of either DNAzyme or control oligonucleotide (ODN) were then added to the cells respectively and incubated at 37°C for 4 h in 5%CO_2_, followed by the addition of complete medium to the wells and further incubation for indicated time.

ATM-specific siRNA (sc-29761) and control siRNAs were purchased from Santa Cruze. siRNAs or a scrambled control siRNA were transfected into the LMP1-positive cells by using Lipofectamine 2000 according to the manufacturer's protocol (Invitrogen).

### NF-κB inhibitor and cell treatments

The specific NF-κB inhibitor Bay11-7082 (Calbiochem) were prepared as a stock solution of 20 mM in dimethylsulfoxide (DMSO, Sigma). Subconfluent cells were treated with the compound at indicated concentrations for the indicated time. The final concentration of DMSO in the culture media was kept less than 0.1% which had no significant effect on the cell growth.

### Western blot analysis

Cells were harvested and washed twice with ice-cold phosphate-buffered saline (PBS), and lysed in the lysis buffer [10 mM Tris–HCl, pH 8.0, 1 mM EDTA, 2% SDS, 5 mM dithiothreitol, 10 mM phenylmethyl sulfonylfluoride, 1 mMNa3VO4, 1 mM NaF, 10% (vol./vol.) glycerol, protease inhibitor cocktail tablet (Roche)] for 30 min on ice and then centrifuged at 15,000×g for 10 min. The supernatant was collected as the whole cell lysates. Protein concentration was determined by BCA Assay Reagent (Pierce). 100 µg of the total proteins from various cell preparations and rainbow molecular weight markers (Amersham Pharmacia Biotech, Amersham, United Kingdom) were separated on SDS polyacrylamide gel and then electrotransferred onto the nitrocellulose membrane. The membranes were blocked with buffer containing 5% non-fat milk in PBS with 0.05% Tween-20 for 2 h, and incubated with different primary antibodies overnight at 4°C. After second wash, the membranes were incubated with anti-rabbit (sc-2004, Santa Cruz) or anti-mouse (sc-2005, Santa Cruz) horseradish peroxidase-conjugated secondary antibody for 1 h at RT and developed with the enhanced chemiluminescence detection kit (ECL; Pierce). The following antibodies were used for Western blotting: mouse LMP1 monoclonal antibody (M0897, DAKO), ATM (sc-23922, Santa Cruz), p-IκBα against Ser-32 of p-IκBα (sc-8404, Santa Cruz), IκBα against the C-terminus of IκBα(sc-371, Santa Cruz), α-tubulin (sc-5286, Santa Cruz), β-actin (sc-8432, Santa Cruz).

### Real-time PCR

Total RNA was isolated from HNE2 and HNE2-LMP1 cells with the TRIzol reagent (Invitrogen) according to the manufacturer's instructions, followed by cDNA synthesis using the SuperScript™ IIRT (Invitrogen). Real-time PCR was performed with Stratagene Mx3000P (Agilent Technologies, Redwood City, CA, USA) using SYBR Green qPCR Supermix-UDG universal PCR Master Mix (Invitrogen). cDNA was subjected to PCR with PCR primers specific for ATM (sense: 5′-CAGCAGCTGTTACCTGTTTG-3′ and anti-sense: 5′-TAGATAGGCCAGCATTGGAT-3′) and LMP1 (sense: 5′- TGACTGGACTGGAGGAGC-3′ and 5′-AGCGATGAGCAGGAGGGT-3′. The reaction was amplified as follows: 40 cycles of 95°C for 15 s and 60°C for 60 s followed by an extension at 72°C for 10 min. PCR reaction with porcine β-actin primers (sense: 5′-CACGCCATCCTGCGTCTGGA-3′, and antisense: 5′-AGCACCGTGTTGGCGTAGAG-3′) was used as an internal control.

### Transient Transfections

Subconfluent proliferating CNE1 cells were co-transfected with increasing amounts of pSG5-B-346-LMP1 LMP1 expressing plasmid (0, 0.5, 1 ug/well) and decreasing amounts of control vector pSG5 (1, 0.5, 0 µg/well) using Lipofectamine 2000 (Invitrogen). Twenty four hours after transfection, the cells were harvested for western blot assay.

### Luciferase Assay

The construct (pLuc-ATM) was cotransfected with the pRL-TK vector (Promega) at a ratio of 10∶1 into cells using lipofectamine2000. Twenty four hours after transfection,the cells were collected and luciferase activity was measured using the Dual Luciferase Assay (Promega, E1910). Briefly, 100 µl of firefly luciferase substrate (LARII) and 20 µl of cell extract were mixed, and the reaction was immediately measured for 10 s. Then 100 µl of Renilla luciferase substrate including an inhibitor for firefly luciferase (Stop & Glow) was added, and light emission was detected for another 10 s interval. The ratio of both measurements (pGL/pRL) represents the relative luciferase activity.

### Bioinformatics analysis

Bioinformatics analysis was performed for the ATM promoter region (GenBank Accession GXP_480587) and three putative NF-κB binding sites were found. The first potential NF-κB binding site was identified at position 107 to 119, on the sense strand (NF1); the second site at position 290 to 320 (NF2) and the third binding site at position 108 to 120 (NF3) on the antisense strand. The sequences of the first and third binding sites were same but on different strands.

### Electrophoretic Mobility Shift Assay (EMSA)

The NF-κB EMSA was performed according to manufacturer's instructions (Pierce). Briefly, 8×10^6^ cells were washed in ice-cold phosphate-buffered saline and the nuclear protein was extracted with the Nuclear Extract Kit (Active Motif). Protein concentration was determined by BCA Assay Reagent (Pierce). Two double-stranded ATM probes corresponding to the NF-κB binding sites in the ATM promoter region were designed as NF1: 5′- GGGCCGGGGAACTCCCTATTTGCC -3′; NF2: 5′- GTGGCGAGGGGAAGCCCGAGGGG -3′. Approximately 20 fmol of biotin labeled ATM probes were added to 10 µg of nuclear extract. The Oct-1 probe containing the sequence 5′- TGTCGAATGCAAATCACTAGAA-3′ was used as a binding control oligonucleotide. The binding reaction was carried out for 20 min at room temperature in 10% glycerol, 60 mM KC1, 1 mM EDTA, 1 mM dithiothreitol, 1 µg/µl poly[dI–dC] and a 200-fold molar excess of unlabeled competitor oligonucleotide (unlabeled probe) or mutated oligonucleotides (NF1 or NF2 with mutaions:5′-GGGCCGAGAAACTCCGTATTTGCC -3′;5′-GTGGCGAGAGACAGCCCGAGGGG-3′) or nonspecific oligonucleotide. For supershift experiments, 1 µg of specific anti-p65 or anti-p50 or anti-p52 antibodies were added to the binding reaction and incubated for 30 min, after which the biotion-labeled probe was added. DNA–protein complexes were separated by 5% non-denaturing polyacrylamide gel.

### Irradiation of cells and FACS analysis

Flow cytometry analysis was performed to study the effect of the ATM-siRNA ± irradiation (IR) on cellular apoptosis. Cells were transfected with the ATM-siRNA or Control-siRNA. 48 h later cells were irradiated with 5 Gy (X ray) and collected for FACS analysis 72 h after culture. Untreated and treated cells were then washed with ice-cold PBS and suspended in 75% ethanol at 4°C overnight. Fixed cells were centrifuged and washed with PBS twice. Before flow cytometry analysis, cells were stained with 50 µg/ml of PI and 0.1% of RNase A in 400 µl PBS in a light-proof tube at 25°C for 30 min. Stained cells were assayed on FACSort (Becton Dickinson) and the cell cycle parameters and the percentage of apoptotic cells (sub-G1 peak) were determined using the CellQuest software program (Becton Dickinson).

### Colony-forming assay

Colony-forming analysis was performed to study the effect of the ATM-siRNA or Control-siRNA or DNAzyme (DZ1) or control oligo (ODN) ± IR treatment on cell proliferation. Briefly, LMP1-positive cells CNE1-LMP1 were transfected with ATM-siRNA or Control-siRNA or DNAzyme (DZ1) or control oligo (ODN). 48 h later 100, 200 or 1000 cells were seeded into the 6-well dishes and cultured for another 24 h. These cells were irradiated with 1 Gy, 2 Gy and 3 Gy X ray, followed by further culturing for 14 days to allow colonies to form. Colonies (>50 cells) on dishes were visualized by staining with 0.0125% crystal violet (w/v in 75% ethanol; Sigma–Aldrich, St. Louis, MO). Plating efficiency was determined as the ratio of the number of colonies divided by the number of cells seeded. Each data point represents the mean of three independent experiments. The data were analyzed using linear-quadratic model [24].

### Animal studies

#### Human nasopharyngeal carcinoma xenograft model in nude mice

5×10^6^ CNE1-LMP1 cells in 0.2 ml of serum-free RPMI 1640 medium were inoculated s.c. into the right flank of six-week-old female BALB/c nude mice (6 mice per group). Tumor volumes were determined according to the formula of length × width × height × (π/6). When the tumor volume reached 60–100 mm3, the animals were injected intra-tumourally with 10 µg of Dz1 or CON with 3 ul FuGene6 (Roche), or saline alone twice/week in an injectate volume of 20 µl. At the end of the experiments (day 18 from the first treatment), mice were sacrificed, tumor tissues stored for histochemical examination. In the groups where the mice were subjected to irradiation treatment (IR), two doses of local irradiation at 5 Gy (^60^Co) were given 24 h after the first two DNAzyme injections.

### Histology and Immunochemistry

Tumours were removed from euthanized mice and fixed in 4% neutral formalin at room temperature for 48 h. Serial tissue sections at 5-µm thick were obtained after embedding in paraffin. Each slide was stained with haematoxylin and eosin (H&E). Tissues sections were stained using an anti-LMP1 mAb (DAKO, Cat#M0897), or NF-kB p65 antibody (Santa Cruz, Cat#8008) at 1∶100 or 1∶ 200 dilution respectively. All sections were examined by light microscopy (Olympus CX31). The expression of LMP1 and P65 were semi-quantitatively analyzed under a light microscope at 40× magnifications. The total visual areas (A) were traced randomly and the total areas (B) of the positive cells in the visual area were determined using an image analyzer (Highspeed Color Image Analyzer SP500; Olympus, Tokyo, Japan). The results were expressed as a percentage of B/A (% positive units).

### Statistical analyses

All the data were expressed as mean ± SD. The SFs of each of the two different treatment cells were compared to the SF for the parent line, separately for each dose level, using one-way ANOVA. Next, SPSS statistical software version 18.0 was used to estimate the parameters of the linear-quadratic model. The statistical difference *p*<0.05 was considered as significant and *p*<0.01 as very significant.

## Results

### LMP1 enhances ATM expression in human nasopharyngeal carcinoma cells

**Figure 1:**
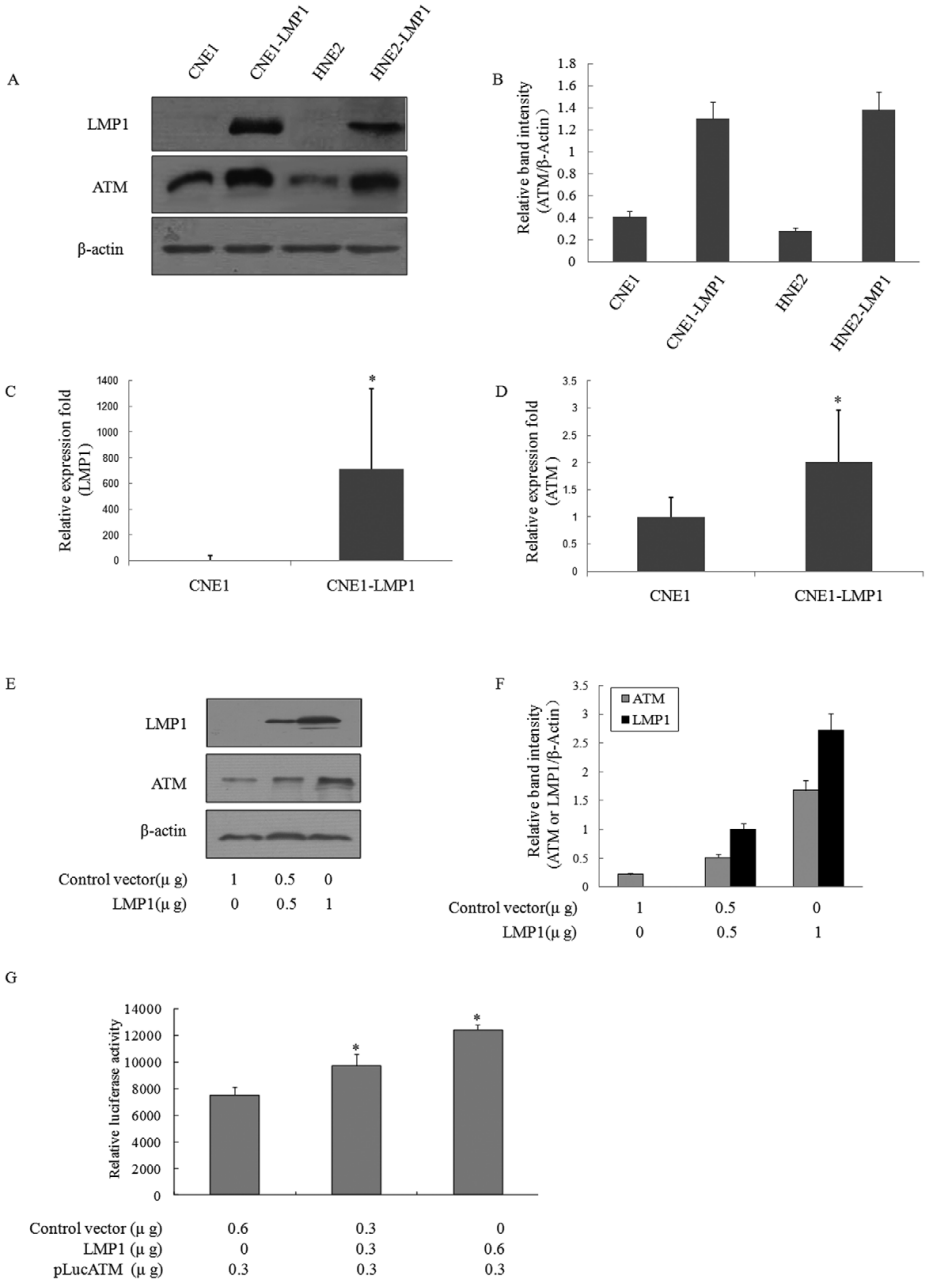
LMP1 enhances ATM expression in human nasopharyngeal carcinoma cells. A, The expression of ATM and LMP1 in CNE1,CNE1-LMP1,HNE2,HNE2-LMP1 cells by western blots. B, Expression level of ATM was estimated by densitometry and presented as a ratio to the loading control β-Actin. C, The expression of LMP1 in CNE1 and CNE1-LMP1 cells by Real-time PCR.D, The expression of ATM in CNE1 and CNE1-LMP1 cells by Real-time PCR. E, CNE1 cells were transiently transfected with increasing amounts of LMP1-expressing plasmid for 24 h and analyzed by Western blots for the expression of ATM, LMP1, and β-actin. F, Expression level of each protein was estimated by densitometry and presented as a ratio to the loading control β-Actin. G, Increasing amounts of LMP1 were cotransfected with pLuc-ATM plasmid into CNE1 cells followed by luciferase assay. (Values are the means ± SD of three replicates, *p<0.05 compared with the control cells).

To study the effect of LMP1 on ATM expression in NPC cells, we used a constitutely expressing LMP1 cell line CNE1-LMP1,HNE2-LMP1 and LMP1-negative cell line CNE1,HNE2. We observed that the ATM expression in LMP1-positive cells was elevated compared to LMP1-negative cells (Fig. 1A). In order to determine if the observed increase in ATM expression was related to an effect of LMP1 at the mRNA level, the ATM expression in CNE1-LMP1 and CNE1 cells were monitored by Real-time PCR. The ATM mRNA level in CNE1-LMP1 cells was also higher than that in CNE1 (Fig. 1D). Further to confirm these findings, we transiently transfected LMP1-expressing plasmid, pSG5-LMP1, into CNE1. As shown in Fig. 1E, the enforced expression of LMP1 in CNE1 cells also increased the ATM protein level. To investigate the regulatory effect of LMP1 on ATM expression, we con-transfected CNE1 cells with an ATM-promoter-luciferase reporter plasmid (pLuc ATM) and the LMP1-expressing plasmid. The results showed that LMP1 could specifically increase the ATM promoter-controlled luciferase activity in a dose-dependent manner (Fig. 1G). Therefore, we conclude that LMP1 could regulate the ATM expression in the NPC cells and this effect might involve a direct interaction between LMP1 and the ATM promoter.

### Inhibition of LMP1 expression by LMP1-specific DNAzyme decreases ATM production

**Figure 2:**
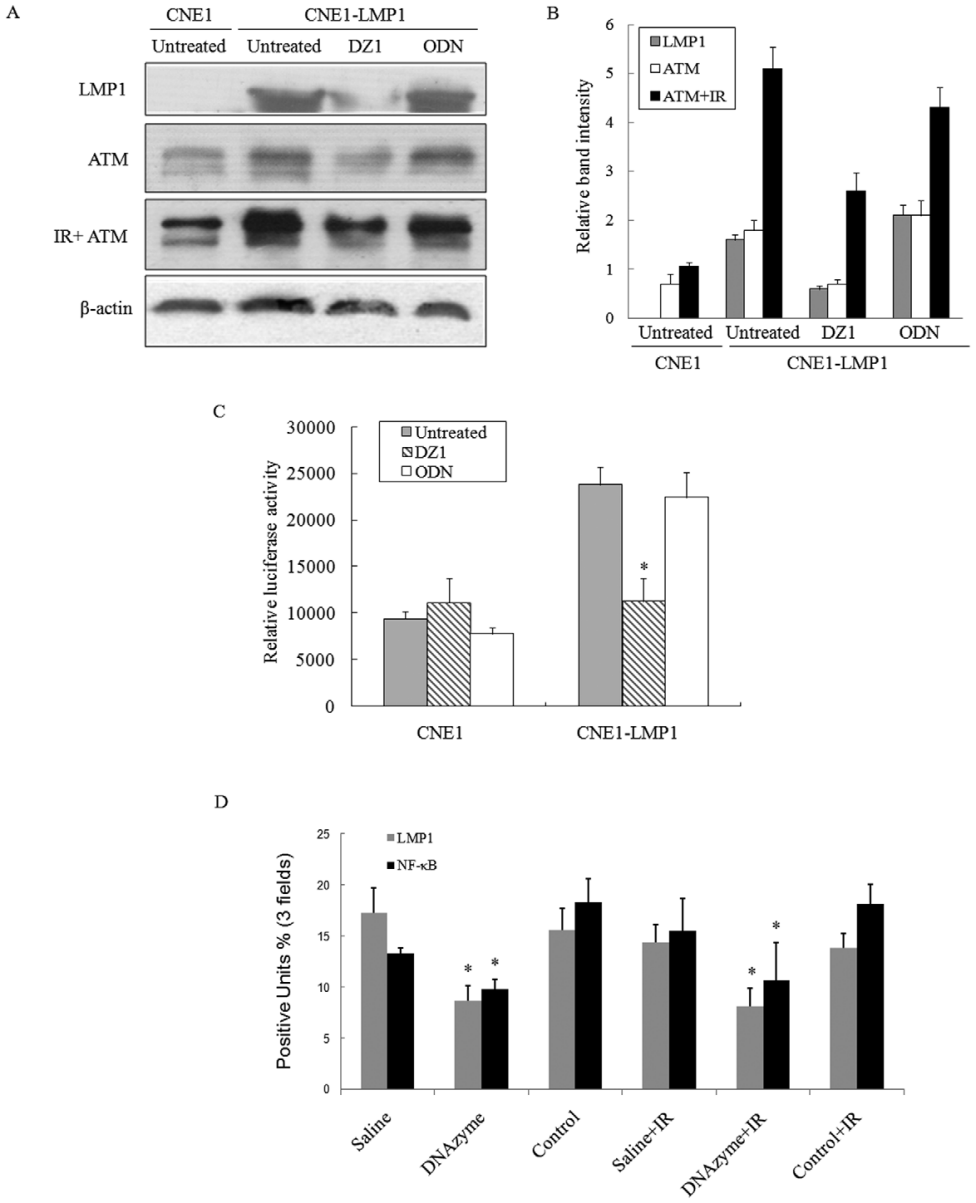
Inhibition LMP1 expression by LMP1-specific DNAzyme decreasing ATM production. A, CNE1 and CNE1-LMP1 cells were grown in six-well plates and CNE1-LMP1 cells were transfected with DNAzyme (DZ1 at 2 µM), or control oligo(ODN), incubated for 24 h, then irradiated at 5 Gy or not. Total cell were havested 1 h later for Western blot using antibodies targeting LMP1 and ATM, β-actin was used as a loading control. B, Expression level of each protein was estimated by densitometry and presented as a ratio to the loading control β-Actin. C, Comparison of transcriptional activation of the ATM promoter in human nasopharyngeal carcinoma cell lines. Transient transfected the constructed plasmid carrying ATM promoter (pLuc ATM) into CNE1 and CNE1-LMP1 NPC cell lines and the cells were transfected with DNAzyme (DZ1 at 2 µM), or control oligo(ODN). The luciferase reporter assays were performed as described in Materials and methods. The relative luciferase activity normalized to the value of Renilla activity. The data represent the mean ± SD of the three independent experiments performed in triplicate. D, Expression of LMP1 and NF-kB in tumor tissues with or without IR. Tumors were removed from euthanized mice and fixed in 4% neutral formalin. Tissues sections were stained using an anti-LMP1 mAb. The expression of LMP-1 was semi-quantitatively analyzed under a light microscope at 40× magnifications. The total visual areas (A) were traced randomly and the total areas (B) of the positive cells in the visual area were determined using an image analyzer. The results were expressed as a percentage of B/A (% positive units).

In order to validate the regulatory effect of LMP1 on the ATM expression, we used the LMP1 targeted DNAzyme to down regulate the LMP1 expression in CNE1-LMP1 and CNE1 cells. When the CNE1-LMP1 cells were treated with a LMP1-targeted DNAzyme, the LMP1 expression was down-regulated as expected, which resulted in the concomitantly decreased level of the ATM expression. Since ATM is an import gene related with IR response we irridiated the cells at 5 Gy. The results showed that after radiation the expression of ATM either in CNE1 or CNE1-LMP1 cells were higher than the cells without radiation. Moreover LMP1-specific DNAzyme can also downregulate the expression of ATM under radiation (Fig. 2A).To further confirm the above observation, we used the ATM-promoter-luciferase reporter system in CNE1-LMP1 cells and showed that while the LMP1 enhanced the ATM promoter activity, the down-regulation of the LMP1 expression by the DNAzyme brought the ATM promoter activity to a basal level (Fig. 2C). This was not observed in CNE1 cells. Together, the observations further confirmed the positive regulatory effect of LMP1 on the ATM expression.

NF-κB is an important transcription factor which is known to activate a lots of genes including DNA damage responsive genes. It has been reported LMP1 can regulate NF-κB expression in vitro. We want to investigate if LMP1 can regulate NF-κB in vivo. To demonstrate the expression of the target gene LMP1 was indeed suppressed in the tumor tissues, immunohistochemical staining was conducted on the cross-sections of paraffin-embedded formalin-fixed tissues from different treatment groups. DNAzyme suppressed the LMP1 expression in these tumors, while immunostaining in the control oligo did not differ from saline control (Fig. 2D). Also to gain if downregulate of LMP1 would influence NF-κB expression or not, NF-κB expression was examined and the results showed that NF-κB was reduced in the DNAzyme and DNAzyme+IR groups in comparison with the saline and control groups (Fig. 2D). These data suggested LMP1 can regulate NF-κB expression whether under irridiation or not.

### NF-κB binds to ATM promoter and regulates ATM promoter activity

**Figure 3:**
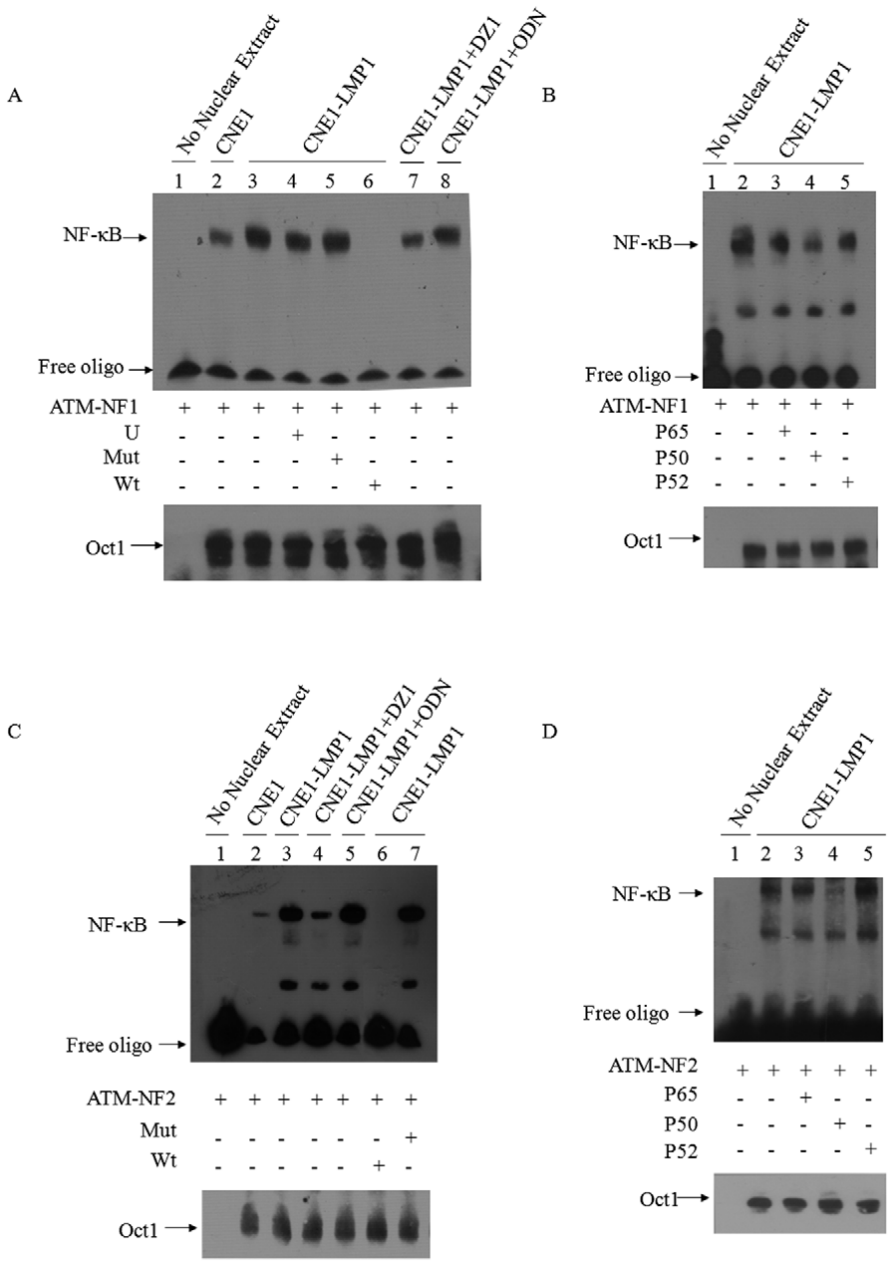
Assay for NF-κB binding to ATM promoter. A, Biotin-labeled wild type ATM-NF1 was incubated with the nuclear extracts from CNE1 (lane 2), CNE1-LMP1 (lanes 3–6) or CNE1-LMP1 treated with Dz1 (lane 7) and CNE1-LMP1 treated with oligonucleotide control ODN (lane 8). A 200-fold excess of noncompetitive unlabelled Stat3 oligonucleotide (U, lane 4), unlabeled mutated ATM-NF1 (mut, lane 5) and unlabeled wild-type ATM-NF1 (wt, lane 6) were included in the respective reactions. Oct1 bands served as loading control. B, Biotin-labeled wild type ATM-NF1 was incubated with the nuclear extracts from CNE1-LMP1 treated with antibodies to p65 (lane 3), p50 (lane 4) and p52 (lane 5). C, Biotin-labeled wild type ATM-NF2 was incubated with the nuclear extracts from CNE1 (lane 2), CNE1-LMP1 (lanes 3) or CNE1-LMP1 treated with Dz1 (lane 4) and CNE1-LMP1 treated with oligonucleotide control ODN (lane 5). Unlabeled wild-type ATM-NF2 (wt, lane 6) and unlabeled mutated ATM-NF2 (mut, lane 7) were included in the respective reactions. Oct1 bands served as loading control. D, Biotin-labeled wild type ATM-NF2 was incubated with the nuclear extracts from CNE1-LMP1 treated with antibodies to p65 (lane 3), p50 (lane 4) and p52 (lane 5).Oct1 bands served as loading control.

Next, we examined the mechanism by which LMP1 activates ATM expression. It has been reported and our data also proved that LMP1 can activate the NF-κB pathway in NPC cells and up-regulate a number of related genes [21], [25], [26]. To elucidate if the LMP1 regulation of ATM is through the NF-κB pathway, we performed bioinformatics analysis and found that there were three putative the NF-κB binding sites in the ATM promoter region (GenBank Accession GXP_480587), which suggested a possible involvement of NF-κB in the LMP-1 mediated augment of ATM expression in NPC cells. The first potential NF-κB binding site was identified at position 107 to 119, on the sense strand (NF1); the second site at position 290 to 320 (NF2) and the third binding site at position 108 to 120 (NF3) on the antisense strand. The sequences of the first and third binding sites were same but **on** different strands. To test whether the NF-κB could bind to the identified sites within the ATM promoter, EMSA assay was performed using the synthetic oligonucleotide corresponding to the consensus NF1 at 107 to 119 bp and NF2 at 290 to 320 bp within the ATM promoter. Gel shift analysis showed that there was a difference of NF-κB binding to the ATM promoter in the presence and absence of the LMP1 expression as tested using the nuclear extracts from CNE1 and CNE1-LMP1 cells (Fig. 3A and 3C). The Dz1 treatment of CNE1-LMP1 resulted in a much weaker binding of NF-κB to the ATM promoter (Fig. 3A and 3C). The specificity of NF-κB binding to the ATM-NF1 and ATM-NF2 was demonstrated by using a 200-fold excess of unlabeled nonspecific oligonucleotide and mutated oligonucleotides and the fact that the use of the unlabeled wild type ATM-NF1 or NF2 oligonucleotides completely competed against the labeled ATM-NF1 or NF2 (Fig. 3A and 3D). The bands of the Oct1 EMSA indicated the equal amount of the nuclear extracts was used in the binding assays. To test which members of NF-κB were involved in the binding, we used the anti-p50, anti-p65 and anti-p52 antibodies to deplete the corresponding NF-κB members in the nuclear extracts. It was showed that the treatment with p65 and p52 did not affect the binding except for p50, suggesting that p50 could be the factor involved in the ATM promoter binding (Fig. 3B and 3D).

**Figure 4:**
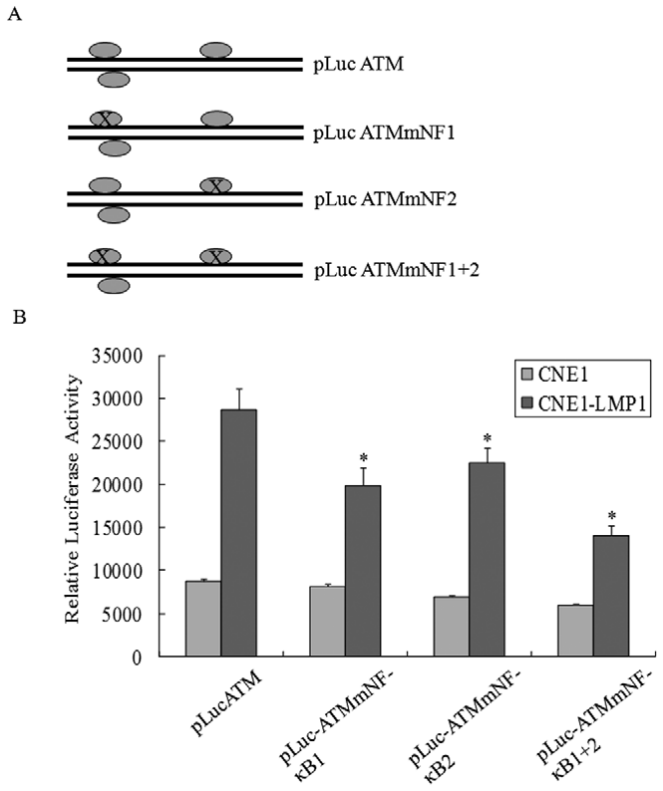
Reporter gene assay for ATM promoter activity. A, Illustration of the reporter gene constructs with either wild-type or mutated NF-κB binding sites in ATM promoter region. B, luciferase repoter assay were performed in CNE1 and CNE1-LMP1 cells transfected with the wild-type or mutant plasmids. The relative luciferase activity normalized to the value of Renilla activity. The data represent the mean ± SD of the three independent experiments performed in triplicate. *p<0.05, compared with the wild-type pLuc-ATM transfected CNE1-LMP1 cells.

To investigate if the NF-κB binding could regulate ATM promoter activity in cells, we constructed a series of luciferase reporter gene constructs driven by the ATM promoter with wild type and mutated binding sites in the pGL3 basic vector (Fig. 4A). Luciferase reporter assays showed that individual mutations at either NF1 (ATMmNF1) or NF2 (ATMmNF2) could decrease the promoter activity by 30%–20%. The mutations at both NF-κB sites in ATMmNF1+2 constructs resulted in nearly 62% loss of the promoter activity in CNE1-LMP1 cells (Fig. 4B). This was not seen in LMP1 negative CNE1 cells. Together, the data supported that the ATM promoter activity could be regulated in NPC cells via a direct NF-κB binding to the promoter region.

### LMP1 promotes ATM expression through the NF-κB pathway

**Figure 5:**
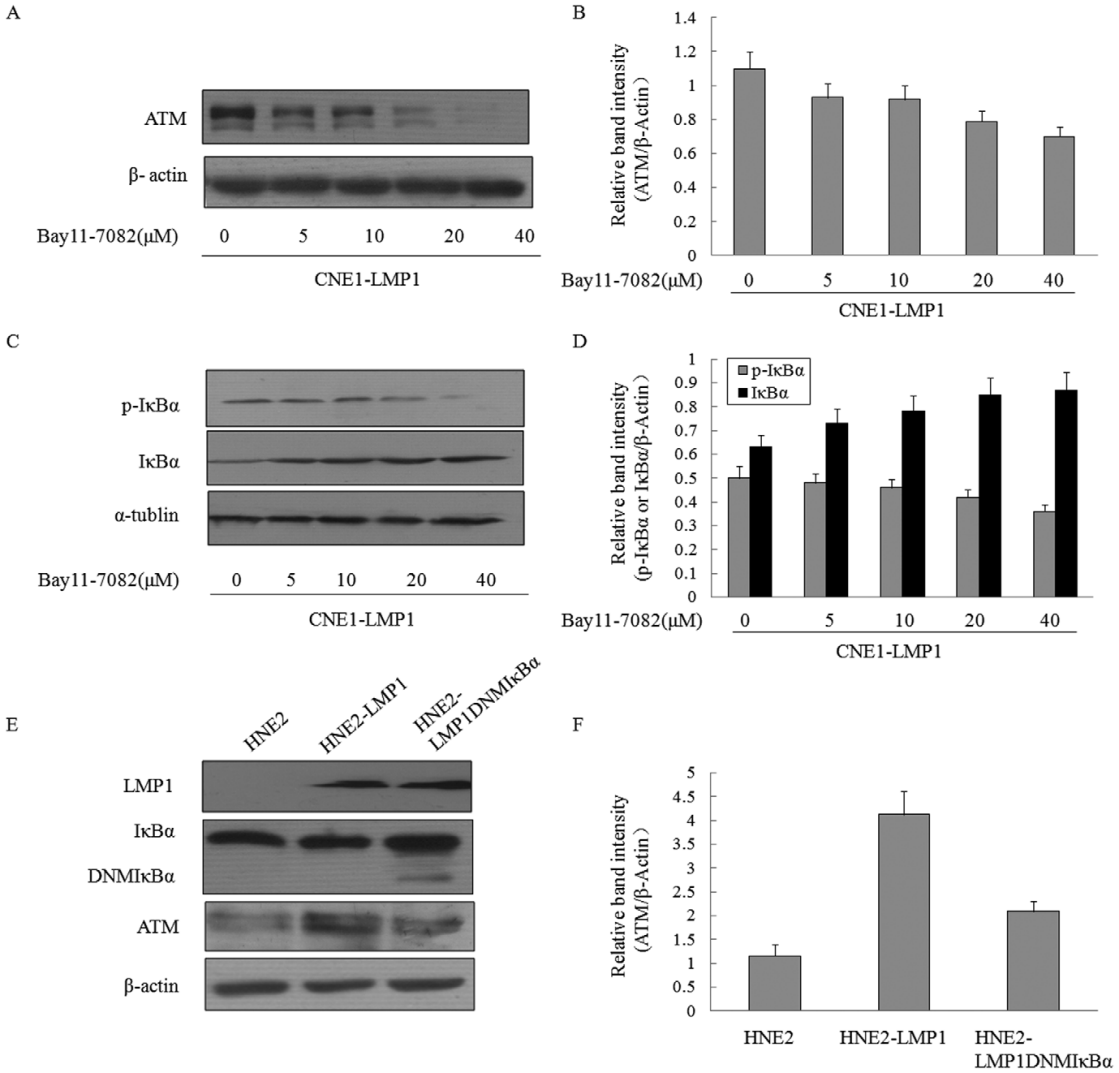
LMP1 mediated increase in ATM expression through the NF-κB pathway. A, CNE1-LMP1 cells were treated with indicated concentrations of Bay11-7082 for 12 h. ATM expression in NPC cells were measured by Western blot and β-actin was used as a loading control. B, Expression level of ATM was estimated by densitometry and presented as a ratio to the loading control β-Actin. C, CNE1-LMP1 cells were treated with indicated concentrations of Bay11-7082 for 2 h and total cell lysate were subjected to Western blots to measure the levels of P-IkBα and IkBα and α-tubulin was used as a loading control. D, Expression level of each protein was estimated by densitometry and presented as a ratio to the loading control β-Actin. E, HNE2, HNE2-LMP1 and HNE2-LMP1 cells expressing DNMIκBα were compared for the LMP1, IkBα, dominant negative IkBα and ATM expression. β-actin was used as a loading control. F, Expression level of ATM was estimated by densitometry and presented as a ratio to the loading control β-Actin.

A specific NF-κB inhibitor, Bay11-7082 [27], was used to investigate if suppression of NF-κB had an impact on the ATM expression. Western blotting showed that the after 12 h treatment of CNE1-LMP1 cells with the NF-κB specific inhibitor [28] resulted in a dose-dependent suppression of ATM expression in CNE1-LMP1 cells (Fig. 5A), which was correlated with a dose-dependent attenuation of IκBα phosphorylation. The decreasing level of phospho-IκBα was accompanied by accumulation of IκBα in cells due to a block of its degradation (Fig. 5C). To further confirm the above observation, a stable NPC cell line expressing dominant-negative mutant of IκBα (DNMIκBα) [29] was used to test the role of NF-κB pathway in regulating ATM expression. DNMIκBα had a deletion of 71 amino acids at the N terminus of IκBα, which could competitively inhibit the activation of NF-κB [30]. DNMIκBα expression could be detected by immunoblotting with an antibody against a peptide mapping at the C-terminus of IκBα (Fig. 5E). As shown by Western blotting, the cell line that had a low level of expression of DNMIkB (HNE2-LMP1-DNMIκBα) exhibited a marginally decreased level of the ATM expression in LMP1 positive cells (Fig. 5E). The data revealed that the ATM expression could be regulated by LMP1 via activation of the NF-κB signaling pathway.

### Inhibition of ATM leads to radiosensitization in LMP1 positive cells

**Figure 6:**
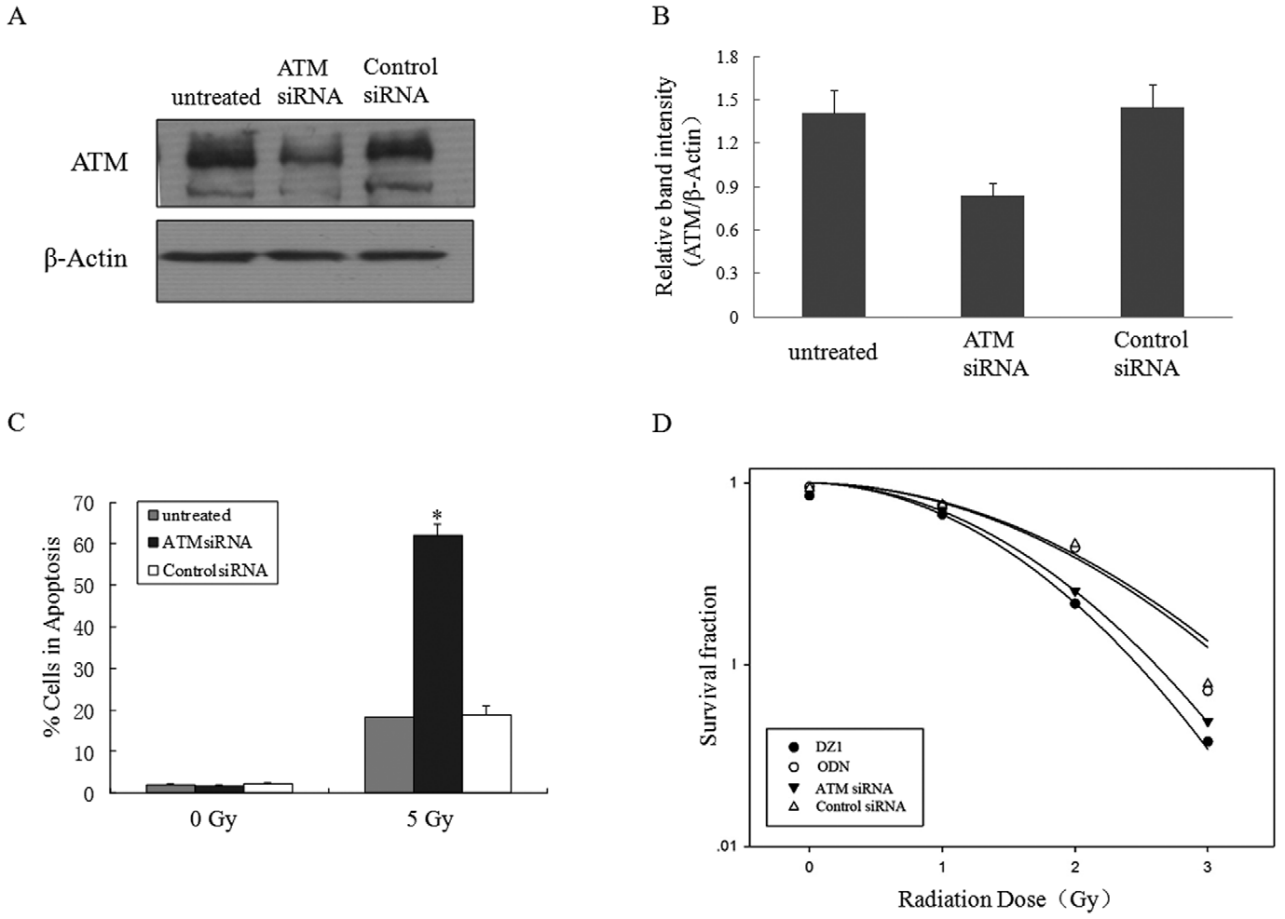
Increase in irradiation (IR)-induced apoptosis and suppression of the colony-formation by inhibition of ATM expression. A, CNE1-LMP1 cells were transfected with ATM-siRNA or Control-siRNA and total cellular protein was extracted 48 h later for Western analysis of the ATM expression. β-actin served as a loading control. B, Expression level of ATM was estimated by densitometry and presented as a ratio to the loading control β-Actin. C, CNE1-LMP1 cells were transfected with ATM-siRNA or control siRNA. 48 h later, the cells were irradiated at 0, 5 Gy and then incubated for 72 h before apoptosis was quantified by FACS. Each data point represents the mean average of three separate determinations, with S.D. values shown. *p<0.05, compared with the Control-siRNA transfected CNE1-LMP1 cells. D, CNE1-LMP1 cells were either transfected with either ATMsiRNA or ControlsiRNA or DNAzyme (DZ1), or control oligo(ODN) and 48 h later were exposed to 0, 1, 2 and 3 Gy of IR and then incubated for 2 weeks before fixation, staining and colony counting. Clonogenic assays were performed in triplicates. The curves were fit to the data using the linear-quadratic model of radiation sensitivity.

To provide further evidence for the role of ATM in regulation of apoptosis in LMP1 positive NPC cells, CNE1-LMP1 cells were treated with 20 pmol of ATM siRNA or control siRNA. A reduction in ATM protein levels was detected at 48 h post-transfection in ATM-siRNA-treated CNE1-LMP1 cells (Fig. 6A). To examine the effect of ATM down-regulation on radiation-induced apoptosis, the siRNA-treated CNE1-LMP1 cells were subjected to an x-ray irradiation. Fig. 6C showed that without radiation (0 Gy) there was no difference of apoptotic rate among the ATM-siRNA-treated, control-siRNA-treated and non-treated CNE1-LMP1 cells. This indicated that ATM loss of function was not sufficient by itself to elicit an apoptotic response. When exposed to irradiation at 5 Gy, nearly 60% of the cells treated with ATM-siRNA underwent apoptosis, while only 20% of cells either treated with control-siRNA or the untreated were in apoptosis (Fig. 6C). The LMP1-regulated radiosensitivity was not seen in the LMP1 negative cell CNE1 (data not shown).

To determine whether inhibition of ATM and LMP1 had any effect on the cell growth under radiation, the clonogenic assay was performed. A linear-quadratic (LQ) model, which has been widely used to analyze the radiation response of cells in the low dose range (0–3 Gy), was applied to the data generation. The clonogenic survival curves of the ATM-siRNA and siRNA control treated cells along with the DNAzyme and ODN treatetd cells were shown in Figure 6D. The survival fractions after exposure to a clinically relevant dose of 2 Gy was defined as SF2 to represent intrinsic radiosensitivity of human tumors. As shown in Table 1, SF2 for the ATM-siRNA and Control-siRNA cells were 0.254±0.006 and 0.462±0.020, respectively. SF2 for the DNAzyme and ODN cells were 0.216±0.009 and 0.440±0.003, respectively. When the dose increased the differences of the surviving fraction between the two cell populations became wider. Together, inhibition of ATM expression could lead to an increased radiosensitivity in LMP1 positive cells.

**Table 1:** Analysis of radiosensitivity in ATMsiRNA and DZ1 transfected cells#.

	α	β	*SF2*	*Surviving fraction (Gy)*			
				*0 Gy,*	*1 Gy*	*2 Gy*	*3 Gy*
ATMsiRNA	0.02703	0.3284	0.2540	0.9230	0.7010	0.2540	0.0486
	±0.174*	±0.078*	±0.006*	±0.029	±0.070*	±0.006*	±0.002*
CONsiRNA	0.0209	0.2155	0.4620	0.9370	0.7600	0.4620	0.0783
	±0.124	±0.044	±0.020	±0.031	±0.069	±0.020	±0.003
DZ1	0.0350	0.3633	0.2167	0.8533	0.6720	0.2167	0.0377
	±0.003※	±0.010※	±0.009※	±0.022※	±0.004※	±0.009※	±0.001※
ODN	0.0260	0.2227	0.4400	0.9500	0.7543	0.4400	0.0717
	±0.002	±0.002	±0.003	±0.013	±0.001	±0.003	±0.001

# Radiobiological parameters were derived from clonogenic survival experiments. The data were generated by SPSS 18.0 for the best fit curves according to the linear-quadratic model. α and β are the initial and final slopes of the linear quadratic survival curves. Clonogenic assays were performed in triplicate. Mean colony numbers of cells relative to plating efficiencies and SE were shown in the table.

*p<0.05, compared with the Control-siRNA transfected CNE1-LMP1 cells (n = 3).

※ *p*<0.05, compared with the ODN transfected CNE1-LMP1 cells (n = 3).

## Discussion

NPC is highly radiosensitive, therefore radiotherapy or radiotherapy in combination with chemotherapy are the main treatment strategies. However, both modalities are usually accompanied with complications and the acquired resistance to the effects of radiotherapy has emerged as a significant impediment to the effective NPC therapy. It has been reported that inhibition of EBV oncoproteins expression including LMP1 enhances the radiosensitivity in EBV-related malignancies both in vitro and in vivo [19], [31]. However, how LMP1 contributes to the radiosensitivity in NPC is still not clear. It is well known now that ATM protein is an important regulator in signaling DNA damage [32]. Gueven et al. found that epidermal growth factor sensitized lymphoblastoid cells to ionizing radiation and that this was accompanied with a reduced level of ATM protein [33]. Down-regulation of ATM protein also sensitizes human prostate cancer cells resulting in an increase in radiation induced apoptosis [34]. In this study, we demonstrated that LMP1, which is expressed in over 75% of NPC cases, could up-regulate ATM expression. Increased level of ATM by LMP1 caused a resistance to irradiation, and decreased level of ATM by siRNA led to a radiosensitization. These data suggested that the interaction between LMP1 and ATM in NPCs may play a critical role in radio-resistance.

Accumulated evidence indicates that the transcription factor NF-κB plays a critical role in cellular protection against a variety of genotoxic agents including IR, and inhibition of NF-κB leads to radiosensitization in radioresistant cancer cells [35]. With microarray analysis, Amundson et al. showed that of 1238 human genes, 48 (3.87%) are inducible by a single dose of irradiation. Interestingly, gene expression profiles of the radioresistant human keratinocyte cell line HK18-IR demonstrate a specific group of stress-responsive genes of which 10–25% are linked with NF-κB activation [35]. Although exact functions of these NF-κB-associated genes are unknown, they are able to influence cell fate through regulating cell cycle and DNA damage repairs. Investigation into these NF-κB target genes is important for elucidation of radiation-induced adaptive resistance. NF-κB was found to be defective in cells from patients with A-T (ataxia-telangiectasia) who are highly sensitive to DNA damage induced by IR and UV lights. Both ATM and NF-κB deficiencies result in increased sensitivity to DNA double strand breaks. Therefore, identification of the molecular linkage between the kinase ATM and NF-κB signaling in tumor response to therapeutic IR will lead to a better understanding of cellular response to IR, and will promise novel molecular targets for therapy-associated tumor resistance.

As to the link between LMP1 and ATM in NPCs, we previously reported that LMP1 could activate different signal transduction pathways that include nuclear factor kappa B (NF-κB), and caused various downstream pathological changes in cell proliferation, anti-apoptosis and metastasis [36], [37], [38], [39]. Several studies demonstrated a correlation of ATM with NF-κB in cell radio-resistance [40]–[41]. In a recent report, a direct interaction between ATM and NF-κB p65 is detected in the resting cells and this interaction is significantly increased with low-dose radiation (LDR) treatment. Inhibition of ATM with caffeine, KU-55933, or siRNA or inhibition of the MEK/ERK pathway can block the LDR-induced NF-κB activation and eliminate the LDR-induced survival advantage [42]. Interestingly, a study of new Rel/NFkappaB regulatory networks showed that NF-κB was involved in regulation of ATM [43]. Here we showed that the ATM expression could be regulated by NF-κB via a direct binding to the ATM promoter and inhibition of NF-κB resulted in reduction of the ATM level. This transcriptional regulation of ATM by NF-κB reveals a novel mechanism that links ATM expression to the radio-resistance in the LMP1 positive NPCs. Thus, it is tempting to suggest that the acquired radio-resistance in NPCs is caused by LMP1 activation of NF-kB, which directly binds to the promoter of the ATM gene and increases the level of ATM expression, leading to a reduced radiosensitivity.

Our data confirmed a positive correlation between LMP1 and ATM expression in NPCs, which appear to be in disagreement with a recent publication [44]. In the study Gruhne et al found that LMP1 could down-regulate the ATM exprssion in a LMP1- transefected B-lymphoma line BJAB, suggesting a reduced DNA repair capacity. However, Bose et al showed that the level of ATM was reduced in NPC clinical smaples, but this reduction was not related to LMP1 [45]. While it would be difficult to reconcile all the differences in relation to the interaction between LMP1 and ATM, it did indicate a complex regulatory network in different cancers, in this case, lymphomas and NPCs. In addition, ATM has been viewed as a tumor suppressor gene that functions at multiple levels involved in cell proliferation, DNA repair, apoptosis and radiosensitivity. It is possible that ATM plays different roles depending on the stages of tumorigenicity and development, and in response to the therapeutic intervention.

In conclution, LMP1 participates in a number of important cellular processes, including radio-resistance in NPCs that could be regulated through the interactions between NF-κB and ATM molecules. This may present a new strategy for an enhanced radiotherapy for NPCs by targeting the LMP1 alone or in combinations with other genes involved in the pathway.
